# Characterizing habitat suitability for a central‐place forager in a dynamic marine environment

**DOI:** 10.1002/ece3.3827

**Published:** 2018-02-09

**Authors:** Dana K. Briscoe, Sabrina Fossette, Kylie L. Scales, Elliott L. Hazen, Steven J. Bograd, Sara M. Maxwell, Elizabeth A. McHuron, Patrick W. Robinson, Carey Kuhn, Daniel P. Costa, Larry B. Crowder, Rebecca L. Lewison

**Affiliations:** ^1^ Stanford University, Hopkins Marine Station Pacific Grove CA USA; ^2^ Southwest Fisheries Science Center Environmental Research Division National Marine Fisheries Service National Oceanic and Atmospheric Administration Monterey Monterey CA USA; ^3^ Department of Parks and Wildlife Kensington WA Australia; ^4^ Institute of Marine Sciences University of California Santa Cruz Santa Cruz CA USA; ^5^ University of the Sunshine Coast Maroochydore Qld Australia; ^6^ Old Dominion University Norfolk VA USA; ^7^ Department of Ecology and Evolutionary Biology University of California Santa Cruz Santa Cruz CA USA; ^8^ Marine Mammal Laboratory Alaska Fisheries Science Center National Marine Fisheries Service National Oceanic and Atmospheric Administration Seattle WA USA; ^9^ Center for Ocean Solutions Stanford University Monterey CA USA; ^10^ Institute for Ecological Monitoring & Management San Diego State University San Diego CA USA

**Keywords:** California current system, California sea lion, distribution, dynamic habitat, telemetry, *Zalophus californianus*

## Abstract

Characterizing habitat suitability for a marine predator requires an understanding of the environmental heterogeneity and variability over the range in which a population moves during a particular life cycle. Female California sea lions (*Zalophus californianus*) are central‐place foragers and are particularly constrained while provisioning their young. During this time, habitat selection is a function of prey availability and proximity to the rookery, which has important implications for reproductive and population success. We explore how lactating females may select habitat and respond to environmental variability over broad spatial and temporal scales within the California Current System. We combine near‐real‐time remotely sensed satellite oceanography, animal tracking data (*n* = 72) from November to February over multiple years (2003–2009) and Generalized Additive Mixed Models (GAMMs) to determine the probability of sea lion occurrence based on environmental covariates. Results indicate that sea lion presence is associated with cool **(**<14°C**),** productive waters, shallow depths, increased eddy activity, and positive sea‐level anomalies. Predictive habitat maps generated from these biophysical associations suggest winter foraging areas are spatially consistent in the nearshore and offshore environments, except during the 2004–2005 winter, which coincided with an El Niño event. Here, we show how a species distribution model can provide broadscale information on the distribution of female California sea lions during an important life history stage and its implications for population dynamics and spatial management.

## 1 INTRODUCTION

Understanding how highly mobile marine predators select and prioritize habitats can be challenging. Despite the proliferation of animal tracking data and near‐real‐time availability of environmental data products, we still have a limited understanding of how environmental heterogeneity can influence the spatial distribution of many species and populations. Habitat models (or species distribution models, SDMs) provide correlative insight into the biophysical features that may drive habitat preference across a wide variety of taxa, scales, and environments (Guisan & Zimmermann, 2000). Habitat models have provided novel tools for assessing and predicting how animals interact with their environment and are increasingly used for ecological and conservation‐relevant research (Barbet‐Massin, Jiguet, Albert, & Thuiller, 2012; Buckley et al., 2010; Dambach & Rödder, 2011; Elith & Leathwick, 2009; Studwell et al., 2017; Zydelis et al., 2011). Most recently, marine SDMs have been used to identify critical habitat of understudied populations, improve our understanding of distributional shifts in habitat under changing ocean conditions, and support commercial and protected species management (Carvalho, Brito, Crespo, Watts, & Possingham, 2011; Eguchi, Benson, Foley, & Forney, 2017; Hazen et al., 2016; Hobday, Hartog, Spillman, Alves, & Hilborn, 2011; Hooker et al., 2011; Skov et al., 2016).

Despite advances in marine SDMs, the complex life histories of many marine species challenge our ability to understand the spatial distributions and patterns for a population during a particular stage or cycle (Ficetola, Pennati, & Manenti, 2013). Modeling habitat suitability for populations of mobile animals with stage‐specific spatial constraints, such as colonial breeders (e.g., seabirds, pinnipeds), has been particularly difficult (Pinaud & Weimerskirch, 2005). As central‐place foragers, these animals are constrained to land during breeding and provisioning stages, that is, nesting colonies, rookery haul‐out (Orians & Pearson, 1979). During this time, habitat preference is likely a function of both prey availability and proximity to the central location (Rosenberg & McKelvey, 1999). Accounting for this place‐based constraint in habitat models is important, as the need to provision offspring directly constrains foraging opportunities, which in turn can have profound implications for the behavior, energetics, and overall reproductive success of individuals.

The California Current System (CCS) is a highly dynamic eastern boundary current that is characterized by oceanographic variability on multiple spatial and temporal scales (Chavez & Messié, 2009; Checkley & Barth, 2009; Schwing, Husby, Garfield, & Tracy, 1991). Here, highly migratory species are known to associate with biophysical features that promote predictable coastal upwelling centers, fronts, and eddies year‐round (Croll et al., 2005; Scales et al., 2017; Yen, Sydeman, Bograd, & Hyrenbach, 2006). California sea lions (*Zalophus californianus*; hereafter, sea lions) are among the most abundant top predators in the CCS (Carretta, Forney, & Oleson, 2011; Villegas‐Amtmann, Atkinson, Paras‐Garcia, & Costa, 2012; Villegas‐Amtmann, Simmons, Kuhn, Huckstadt, & Costa, 2011), and populations have continued to grow under the Marine Mammal Protection Act (Carretta et al., 2016; Lowry, Melin, & Laake, 2017; Melin, DeLong, & Siniff, 2008; Roman et al., 2013). In the United States, sea lions breed primarily at four of the Channel Islands in southern California (Santa Barbara Island, San Clemente Island, San Miguel Island, and San Nicolas Island). San Miguel Island and San Nicolas Island are the two largest rookeries, accounting for approximately 90% of the pups produced in the United States (Lowry et al., 2017).

The distribution, foraging ecology, and reproductive strategies of sea lions have evolved under the influence of short‐term (i.e., upwelling) and long‐term (i.e., El Niño Southern Oscillation) changes in ocean conditions (Melin et al., 2008; Weise & Harvey, 2008). Throughout the summer breeding months, animals remain close to the rookeries. During the nonbreeding season (August–May), demographic groups spatially segregate. Males disperse from the rookery and are free to exploit productive habitats throughout the CCS (Melin, Delong, Thomason, & Vanblaricom, 2000), while adult females are central‐place foragers for the entirety of the 10‐ to 11‐month lactation period. During this time, females must attend to their young, alternating periodic trips at sea (1–7+ days) with time on land to nurse their pups (McHuron et al., 2016; Melin et al., 2000). Because they are nonmigratory, females are vulnerable to prolonged changes in their foraging environment (Melin et al., 2008). The ability to locate suitable habitat close to the rookery is critical to pup survival (Costa, 2007; Melin et al., 2000; Ono, Boness, & Oftedal, 1987). Suboptimal conditions reduce prey availability, requiring females to alter foraging and attendance patterns. In extreme events, protracted changes to prey distribution and abundance have led to reproductive failures and long‐term population affects (DeLong et al., 1991; Kuhn & Costa, 2014; Lowry et al., 2017; McClatchie et al., 2016; Melin et al., 2008; Trillmich et al., 1991).

Moreover, a majority of sea lion prey items are commercially important species (e.g., northern anchovy (*Engraulis mordax*), sardine (*Sardinops sagax*), Pacific hake (*Merluccius productus*), jack mackerel (*Trachurus symmetricus*), Pacific mackerel (*Scomber japonicus*), and market squid (*Doryteuthis opalescens*)) (Lowry & Carretta, 1999; Lowry, Stewart, Heath, Yochem, & Francis, 1991; Orr, VanBlaricom, DeLong, Cruz‐Escalona, & Newsome, 2011; Weise & Harvey, 2008). A depletion of foraging resources near the colony can lead to increased spatial overlap with fisheries, leading to both direct competition and indirect biological interactions (NMFS, 1997; Weise & Harvey, 2005). Such conflicts include human‐related injuries (Goldstein, Johnson, Phillips, Hanni, & Fauquier, 1999), depredation (loss of commercial and recreational fish), incidental capture in fisheries or bycatch, and entanglement in fishing gear (Beeson & Hanan, 1996; Carretta & Chivers, 2004; Stewart & Yochem, 1987). Many of these interactions are complex and have been difficult to manage (Arthur et al., 2017; Lewison et al., 2015; Maxwell et al., 2015). In particular, bycatch of marine mammals in fisheries that overlap with their foraging grounds has been identified as a critical management issue for the United States fisheries (Beeson & Hanan, 1996). Bycatch mitigation, however, requires a robust understanding of sea lion habitat use and distribution.

While a robust body of literature has documented the biology, ecology, and physiology of female California sea lions (Antonelis, Stewart, & Perryman, 1990; Costa, 1991; Feldkamp, DeLong, & Antonelis, 1989; McDonald & Ponganis, 2013; McHuron et al., 2016; Melin et al., 2000; Villegas‐Amtmann et al., 2011, 2012), these studies focused on foraging characteristics such as dive behavior, trip length, and duration of individuals from a single rookery or in response to El Niño events (Antonelis et al., 1990; Costa, 2007; Melin, Orr, Harris, Laake, & DeLong, 2012; Melin et al., 2008; Ono et al., 1987; Sydeman & Allen, 1999; Trillmich et al., 1991). Although aspects of at‐sea habitat use have been explored (Kuhn & Costa, 2014), our ability to broadly identify suitable foraging habitat of this central‐place forager has been limited. This is in part because the importance of prey species in sea lion diet fluctuates seasonally and annually, making direct observations that coincide with prey distribution difficult (Lowry et al., 1991; Melin et al., 2012; Orr et al., 2011). Because we currently lack the fine‐scale resolution required to correlate foraging habitat with prey distribution, we must rely on the oceanographic processes that serve as proxies to prey distribution (Arthur et al., 2017; Bost et al., 2009).

Here, we couple a multiyear tracking data set with near‐real‐time environmental data to quantitatively characterize and predict the spatial extent of habitat suitability of lactating female sea lions from the two main rookeries in the CCS. We examine broadscale habitat use using satellite tracking data from 72 lactating sea lions to elucidate the biophysical relationships associated with habitat preference. We then develop predictive models of habitat to explore how accessibility and use changes among years. Our findings demonstrate the utility of a marine SDM to identify changes in habitat use of a central‐place forager. Given the importance of habitat use of breeding and provisioning females on population‐level processes, our model can be used to connect at‐sea distribution shifts to changes in population trends, as well as inform species protection and fisheries bycatch management efforts.

## 2 MATERIALS AND METHODS

### 2.1 Data sets and tagging methodology

The movement and distribution of California sea lions were examined using ARGOS tracking data from 72 adult lactating females, tagged in November of 2003–2009. Seventeen females were tagged from San Miguel Island (34.0°N, −120.4°W) and 55 from San Nicolas Island (33.3°N, 119.5°W; Table 1). The tracking period for each year lasted between November and February, although the tracking duration of individual animals did not necessarily span this entire time period. All analyses presented were restricted to this time period. Description of animal capture and instrumentation are provided in Kuhn and Costa (2014) and McHuron et al. (2016).

**Table 1:** Biometric data for the 72 adult female California sea lion trips used in the GAMM, from 2003 to 2009. A foraging trip was defined as the time at sea between haul‐out periods (Villegas‐Amtmann et al., 2008). Maximum trip distance from colony refers to the distance between the colony and the farthest away an animal traveled

Tag ID	Tagging location	Start date of trip	Date end of trip	Trip duration (days)	Max distance from colony (km)	Total trip distance (km)
2103020	San Nicolas	11/16/03	11/24/03	8	119.43	223.3
2103029	San Nicolas	11/14/03	11/20/03	6	80.24	80.24
2103030	San Nicolas	11/18/03	11/22/03	4	37.92	79.15
2103033	San Nicolas	12/16/03	12/26/03	10	102.39	144.4
2103035	San Nicolas	12/3/03	12/9/03	6	78.78	95.11
2103036	San Nicolas	11/14/03	11/16/03	2	8.08	8.08
2103037	San Nicolas	11/18/03	11/23/03	5	64.58	92.85
2104001	San Nicolas	11/20/04	11/28/04	8	57.24	77.36
2104003	San Nicolas	11/2/04	11/16/04	14	456.95	895.12
2104004	San Nicolas	11/3/04	11/12/04	9	135.78	178.93
2104005	San Nicolas	10/31/04	11/10/04	10	100.06	181.96
2104006	San Nicolas	2/23/05	3/8/05	13	84.37	112.27
2104007	San Nicolas	11/24/04	12/10/04	16	168.62	230.69
2104008	San Nicolas	11/13/04	11/22/04	9	208.75	432.93
2104010	San Nicolas	11/1/04	11/9/04	8	95.77	160.51
2104011	San Nicolas	11/9/04	11/18/04	9	49.08	65.22
2104012	San Nicolas	11/2/04	11/12/04	10	126.25	238.12
2105019	San Miguel	12/17/05	12/23/05	6	163.08	227.6
2105020	San Miguel	12/14/05	12/19/05	5	50.13	101.04
2105021	San Miguel	1/5/06	1/15/06	10	369.27	746.64
2105022	San Miguel	11/18/05	11/25/05	7	136.5	273.04
2105023	San Miguel	1/30/06	2/7/06	8	210.3	264.3
2105024	San Miguel	12/29/05	1/5/06	7	105	222.76
2105025	San Miguel	1/8/06	1/13/06	5	165.75	273.12
2105026	San Miguel	1/11/06	1/19/06	8	355.4	705.26
2105027	San Miguel	1/5/06	1/11/06	6	47.8	77.29
2105028	San Miguel	12/21/05	12/29/05	8	321.62	537.21
2105029	San Nicolas	12/6/05	12/11/05	5	40.75	125.31
2105030	San Nicolas	12/13/05	12/25/05	12	255.98	447.27
2105031	San Nicolas	12/26/05	1/3/06	8	194.02	394.32
2105032	San Nicolas	12/2/05	12/7/05	5	49.22	129.57
2105033	San Nicolas	12/5/05	12/10/05	5	13.05	75.43
2105034	San Nicolas	12/6/05	12/12/05	6	8.02	62.97
2105035	San Nicolas	11/17/05	11/23/05	6	57.54	115.6
2105036	San Nicolas	12/25/05	12/31/05	6	143.47	263.84
2105037	San Nicolas	12/3/05	12/9/05	6	78.43	164
2105038	San Nicolas	12/28/05	1/6/06	9	100.79	177.77
2105039	San Nicolas	12/5/05	12/9/05	4	57.93	67.09
2105040	San Nicolas	12/7/05	12/23/05	16	226.93	275.41
2106001	San Nicolas	11/11/06	11/20/06	9	255.41	493.04
2106002	San Nicolas	11/3/06	11/10/06	7	124.96	223.36
2106003	San Nicolas	11/22/06	12/10/06	18	340.42	582.47
2106004	San Nicolas	11/4/06	11/14/06	10	96.23	228.58
2106005	San Nicolas	11/7/06	11/19/06	12	158.29	352.43
2106006	San Nicolas	11/20/06	11/25/06	5	34.05	69.54
2106007	San Nicolas	11/21/06	11/28/06	7	127.12	253.85
2106008	San Nicolas	11/5/06	11/10/06	5	5.02	8.31
2106009	San Nicolas	12/12/06	12/21/06	9	117.92	231.82
2106010	San Nicolas	11/15/06	11/29/06	14	218.31	484.83
2106011	San Nicolas	11/8/06	11/14/06	6	65.82	65.82
2106012	San Miguel	12/17/06	12/24/06	7	133.73	238.52
2106014	San Miguel	12/15/06	12/22/06	7	195.4	349.16
2106015	San Miguel	11/27/06	12/11/06	14	387.18	796.15
2106016	San Miguel	11/11/06	11/19/06	8	92.87	179.56
2106018	San Miguel	12/3/06	12/8/06	5	67.09	178.68
2106020	San Miguel	11/21/06	11/27/06	6	80.47	176.58
2106021	San Miguel	11/14/06	11/19/06	5	137.39	251.58
2107009	San Nicolas	11/21/07	11/27/07	6	121.95	240.23
2107010	San Nicolas	12/22/07	1/4/08	13	108.12	213.83
2107011	San Nicolas	12/31/07	1/6/08	6	41.3	84.56
2107012	San Nicolas	1/4/08	1/15/08	11	502.15	876.47
2107013	San Nicolas	11/28/07	12/8/07	10	243.75	498.97
2107014	San Nicolas	1/12/08	1/18/08	6	158.89	299.49
2107015	San Nicolas	11/11/07	11/19/07	8	74.07	134.17
2107016	San Nicolas	12/28/07	1/9/08	12	456.12	838.08
2107017	San Nicolas	11/25/07	12/5/07	10	248.24	509.06
2108001	San Nicolas	12/12/08	12/18/08	6	94.72	151.28
2108002	San Nicolas	12/14/08	12/21/08	7	102.9	218.35
2108003	San Nicolas	11/18/08	11/30/08	12	415.27	765.57
2108005	San Nicolas	11/19/08	11/29/08	10	185.83	397.52
2108006	San Nicolas	11/18/08	11/26/08	8	123.1	249.3
2108010	San Nicolas	12/18/08	1/10/09	23	120.26	157.86

All data processing and analyses were carried out in the R environment, version 3.3.0 (R Core Team, 2016).

### 2.2 Track filtering and trip identification

Bayesian state–space modeling techniques were used as a filtering method to account for location error (Bailey et al., 2008, 2012; Breed, Costa, Goebel, & Robinson, 2011; Breed, Costa, Jonsen, Robinson, & Mills‐Flemming, 2012; Jonsen, Flemming, & Myers, 2005). In order to reduce autocorrelation of at‐sea locations, final position estimates along each track were generated at 24‐hr intervals resulting in one position per day (Austin, Bowen, & McMillan, 2004; Kuhn & Costa, 2014). All points over land were removed from final track locations. A foraging trip was defined as the time at sea between haul‐outs from the rookery (Villegas‐Amtmann, Costa, Tremblay, Salazar, & Aurioles‐Gamboa, 2008). Kernel density analyses of at‐sea locations were used to determine the home range (95%) and core (50%) for each rookery during the entire tracking period (*adehabitatHR* package, Calenge (2011).

### 2.3 Quantifying space use

The efficacy of SDMs often depends on the quality and quantity of presence data points as well as the method of selection of absence points or background data (“pseudoabsences”; e.g., random, environmentally, or spatially stratified; Barbet‐Massin et al., 2012). Here, we created suitable habitat models using presence‐only tracking data and generated pseudoabsences from correlated random walk models (CRWs; Aarts, MacKenzie, McConnell, Fedak, & Matthiopoulos, 2008; Hazen et al., 2016; Willis‐Norton et al., 2015). Such absences represent a theoretical null model where sea lions would travel independent of environmental parameters. Comparison of environmental conditions along sea lion tracks and CRWs can test whether animals are selecting habitat based on specific oceanographic variables (Willis‐Norton et al., 2015).

Owing to the difficulties of quantifying habitat suitability for central‐place foragers (Aarts et al., 2008; Matthiopoulos, Harwood, & Thomas, 2005) and of parameterizing correlated random walks that can accurately approximate their movements, this approach has not been widely implemented. Here, we explore the utility and parameterization of CRWs for a central‐place forager in modeling habitat suitability over broad spatial and temporal scales. Random walk trajectories were simulated using the *adehabitatLT* package in R (Calenge, 2015). Due to the nature of female sea lion movements, CRWs were simulated by trip and by trip phase (i.e., incoming and outgoing portions of each foraging trip). A minimum of 10 CRW simulations were generated per trip and were allowed to move unconstrained except for on land, in which a new location along the trip length was sampled with replacement. Each simulation started at the first observed trip latitude/longitude location and was built iteratively so that the simulated movement was sampled from a normal distribution (e.g., Figure S1). Each simulation maintained the same relative distance, turning angle, and duration in time between successive locations (Calenge, Dray, & Royer‐Carenzi, 2009).

Each simulated trip was weighted based on how closely it resembled the actual sea lion trip. The weight value was calculated as the normalized difference between the actual trip and simulated trip length distance, summed with the normalized distance in net angular displacement of the sea lion and CRW track (Hazen et al., 2016).

(1) $$\begin{array}{cc} \text{Weight}=\; & 2*\left({\text{distance}}_{\mathrm{track}}-{\text{distance}}_{\mathrm{CRW}}\right)/{\text{distance}}_{\mathrm{track}}\\ & +\left({\text{angle}}_{\mathrm{track}}-{\text{angle}}_{\mathrm{CRW}}\right)/90^{\circ } \end{array}$$

The higher the weight value, the more dissimilar the CRW to the actual trip. Such weighted values provided a means of ensuring that the CRWs were at an appropriate distance and direction as compared to the actual movements of sea lions. Simulated trips with weighted values in the upper quartile, and those that crossed land, were removed to ensure that the CRWs were representative of possible movements, distributions, and habitats that sea lions could have encountered (Hazen et al., 2016).

### 2.4 Remotely sensed oceanographic data

Remotely sensed environmental data were obtained for both sea lion and CRW tracks using Xtractomatic (Simmons, 2016). The data sets included time series of sea surface temperature (SST), surface chlorophyll‐a concentrations (Chl‐a), surface winds (v‐component, for upwelling‐favorable conditions), mean sea‐level anomaly (SLA), SST standard deviation (SST SD), SLA standard deviation (SLA SD), bathymetry, and rugosity (bathymetric standard deviation, bathymetry SD; Table 2, see Table S1 for data references). For each oceanographic parameter, a mean value was calculated based on the mean latitude and longitude error 1° longitude × 1° latitude × 1–8 day intervals and centered at the position of each daily SSM‐interpolated sea lion position (Willis‐Norton et al., 2015). The distance of each satellite location from the colony was calculated using great circle distances to account for the Earth's curvature (Kappes et al., 2010).

**Table 2:** List of environmental variables and hypothesized influence on adult female sea lion habitat selection

Variable	Hypothesized mechanistic link
Sea Surface Temperature (SST)	Description of thermal regime
Sea Surface Temperature Standard Deviation (SST SD)	Mesoscale thermal structure
Chlorophyll‐a (Chl‐a)	Proxy for primary productivity
Eddy kinetic energy (EKE)	Index of mesoscale convergence and divergence, prey retention
Mean sea‐level anomaly (SLA)	Index of mesoscale features
Mean sea‐level anomaly Standard Deviation (SLA SD)	Index of mesoscale variability
Wind (v‐component)	Upwelling‐favorable winds
Bathymetry	Depth to seafloor
Bathymetry Standard Deviation (Bathymetry SD)	Roughness of seafloor
Distance from colony	Index of movement from rookery

We also explored mesoscale structure in surface currents using eddy kinetic energy (EKE), which was calculated from geostrophic current components as follow (Cayula & Cornillon, 1992):

$$\text{EKE}=\frac{1}{2}\left(u^{2}+v^{2}\right)$$

Transformations of variables were explored to ensure data were normally distributed. A logarithmic transformation was required for Chl‐a and EKE. A square root transformation was applied to bathymetry SD.

### 2.5 Generalized additive mixed models

Generalized Additive Mixed Models (GAMMs) were used to quantify the statistical correlation between oceanographic parameters and sea lion spatial distribution (Redfern et al., 2006). GAMMs allow for multiple nonlinear relationships between a response variable and its covariates in a semiparametric manner (Hastie & Tibshirani, 1990; Su, Sun, Punt, Yeh, & DiNardo, 2011; Wood, 2006). The GAMMs link the environmental covariates to animal presence/absence with individual as a nested variable. Specifically, GAMMs were fit with a binomial distribution, logit link function, and random effect of individual sea lion. To avoid pseudoreplication, only one trip per individual (the trip with the most number of locations) and one CRW of that trip (randomly selected) were used in the models. GAMMs were run using the *gamm4* package (Wood & Scheipl, 2013).

Because the main focus of this study is on the broadscale habitat use of lactating female sea lions within the CCS, we chose not to run separate models by rookery, but rather to run one model for all individuals. Results provide information on the population‐level habitat associations for the two largest California sea lion rookeries in the CCS.

### 2.6 Model performance metrics

Candidate models were generated based on hypothesized combinations of environmental covariates (Table 2). All variables were tested for multicollinearity using Generalized Variance Inflation Factors (Zuur, Ieno, Walker, Saveliev, & Smith, 2009). The model with the lowest Akaike's Information Criterion (AIC) and highest receiver operating curve (ROC) area under the curve (AUC) statistic was run 40 times with a 1:1 ratio of randomly chosen simulated tracks for each foraging trip (outgoing and return) to examine variability. Model validation using ROC curves and AUC statistics was calculated using the *ROCR* package in R (version 1.0‐7; Sing, Sander, Beerenwinkel, & Lengauer, 2015).

### 2.7 Habitat models and predictive surfaces

Predictive surfaces were generated daily and were fit over a set of time‐matched environmental data that corresponded to each November–February satellite tracking period between 2003 and 2009. The spatial resolution of each predictive surface was set to 0.25°, the lowest common resolution of environmental data (Table S1). Daily surfaces were averaged for each November–February period, generating a total of six winter habitat maps. Relative habitat suitability was scaled from 0 (unsuitable) to 1 (highly suitable). Cumulative mean and standard error (SE) suitability maps show the variability associated with model predictions.

## 3 RESULTS

### 3.1 General habitat use

**Figure 1:**
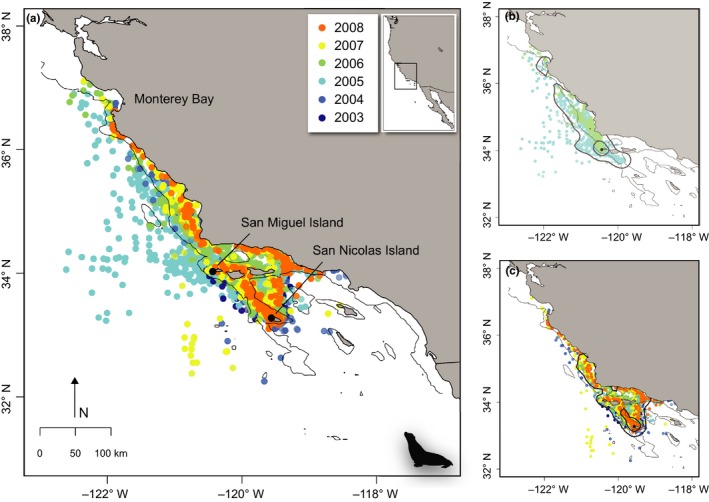
(a) Daily locations from all foraging trips of lactating female California sea lion tracks (*Zalophus californianus, n* = 72), from November to February 2003–2009 (color‐coded by year of deployment) displaying one location per day. Distribution of tracks by colony: (b) San Miguel (*n* = 17) and (c) San Nicolas Islands (*n* = 55). Deployment locations shown as black circles. The 500‐m isobaths are shown in light gray, and the 50% and 95% kernel density utilizations for each colony are shown in dark gray

From 2003 to 2009, adult female sea lions were tracked for 14–131 days (mean 56.9 days ± 24.6 SD). The average number of foraging trips per female was 13.3 ± 6.9 SD trips, with a mean trip duration of 8.4 days (±3.6 days SD) (Table 1). The maximum straight‐line trip distance from the rookery ranged from 5.0 to 502.2 km (mean 149.4 km ± 115.0 km SD). Dispersal primarily extended north/northwest of each colony (San Miguel and San Nicolas islands, Figure 1a); however, core areas of use remained closest to the colonies (50% UD, Figure 1b,c). While most individuals favored nearshore habitat from the Southern California Bight and along the mainland coast, several individuals from each colony were tracked offshore into waters greater than 500 m depth (Figure 1b,c).

### 3.2 Model predictions

**Figure 2:**
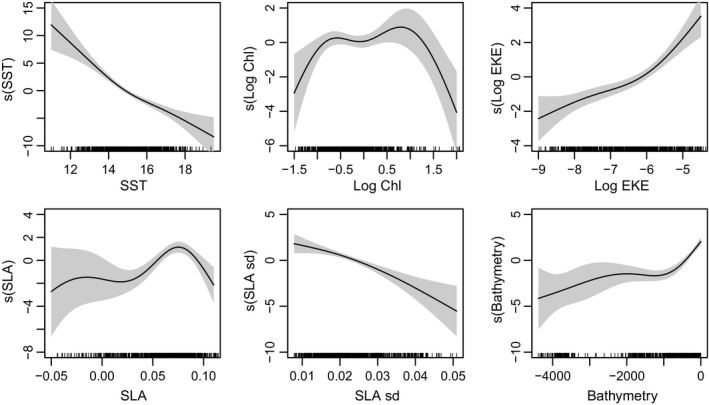
GAMM partial plots showing the relative habitat suitability for adult female California sea lions in response to: sea surface temperature (SST, °C), Chlorophyll‐a (Chl, log mg/m^3^), eddy kinetic energy (EKE, cm^2^/s^2^), sea‐level anomaly (SLA, cm), sea‐level anomaly standard deviation (SLA SD, cm), and bathymetry (m). Gray shading represents the 95% confidence intervals for the fitted relationships

The best‐fitting model included SST, Chl‐a, bathymetry, EKE, SLA, and SLA SD and explained 46% (*R* ^2^ = .46) of the total deviance (Table 3) with an AUC = 0.91 (Figure S2). Partial response curves (Figure 2) show that the probability of sea lion occurrence was greatest with cool SST (<14°C), productive waters (i.e., Chl‐a ranging from −0.5 to 1.0 mg/m^3^), and shallow depths (<500 m below sea level). Sea lions were also associated with increased SLAs (0.05–0.1 cm) and EKE, while SLA SD (i.e., index of mesoscale variability) was negatively associated with sea lion occurrence. Distance from the colony was considered in a competing candidate model, but surprisingly was a less important predictor of sea lion habitat than bathymetry. Overall, SST, EKE, and bathymetry were the most consistently significant predictors of sea lion habitat (Table 3).

**Table 3:** Selection diagnostics from the final Generalized Additive Mixed Model (GAMM). Model was run 40 times to examine the number of times a variable was significant (*n*‐significant). All variables represent a *p* value <.001. Values listed as follow: mean (min – max)

Variable	Effective degrees of freedom (edf)	Chi‐squared	*n*‐significant (*n*/40)
SST	2.7 (1.0–3.8)	160.4 (120.6–198.8)	40
Chl‐a	2.9 (1.0–3.9)	20.0 (1.5–42.3)	24
EKE	3.3 (1.0–3.8)	57.3 (25.1–80.7)	40
SLA	3.1 (1.0–3.9)	39.3 (19.0–70.1)	38
SLA SD	1.2 (1.0–3.5)	27.0 (4.9–71.7)	33
Bathymetry	3.6 (3.3–3.9)	146.2 (106.5–183.2)	40

*R* ^2^ = .46 (.36–.54).

AIC = 864.92 (712.61–1014.59).

AUC = 0.91 (0.88–0.93).

**Figure 3:**
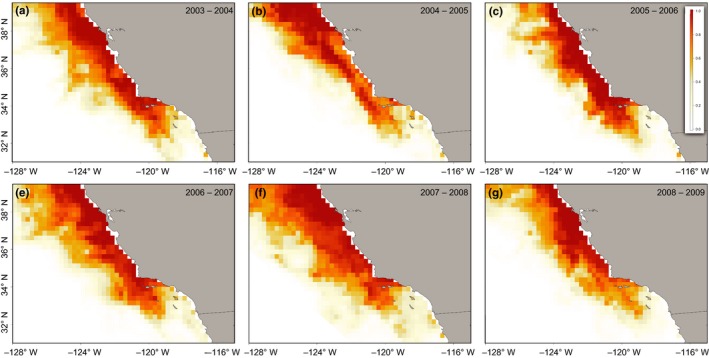
Spatial habitat predictions of adult female California sea lions by year. Maps show relative habitat suitability for female California sea lions during foraging trips, based on environmental data from November to February, from 2003 through 2009. Suitability is scaled from 0 (unsuitable) to 1 (highly suitable)

**Figure 4:**
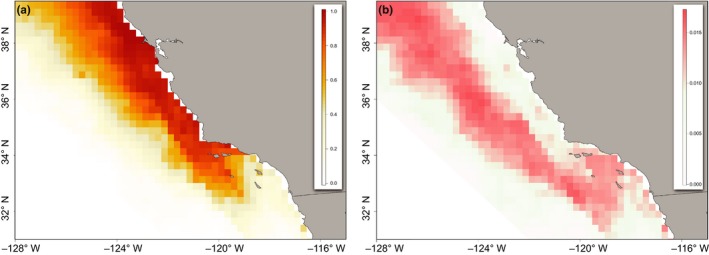
Spatial habitat predictions of adult female California sea lions, averaged over all tracking periods (November–February), from 2003 to 2009. (a) Mean spatial prediction of relative habitat suitability for female California sea lions, from November to February 2003–2009, based on a suite of environmental data. Suitability is scaled from 0 (unsuitable) to 1 (highly suitable); (b) Standard Errors in spatial prediction from November to February 2003–2009. The spatial resolution of predictive surfaces was set to 0.25°, the lowest common resolution of environmental data

The combined influence of these biophysical parameters is evident in the broadscale spatial habitat predictions. Throughout each winter, highly suitable habitats were evident in the near to offshore environments. Available habitat was identified along the California coast, from the northern Channel Islands, up through Monterey Bay. Two exceptions were the winters of 2004–2005 and 2008–2009. During both time periods, habitat suitability diminished within the Southern California Bight (Figure 3b,f). In the winter of 2004–2005, habitat suitability shifted offshore and north of Monterey Bay (Figure 3b). In the winter of 2008–2009, suitability was most concentrated along the nearshore central California coast. Prediction errors across all sampled years were highest in offshore and within the Southern California Bight (Figure 4b).

## 4 DISCUSSION

Characterizing habitat associations for a mobile marine predator can be challenging for animals whose movement patterns change among life stages. Modeling habitat suitability for spatially constrained foragers is a particularly complex exercise because the options to respond to their environment are limited (Kappes et al., 2010; Pinaud & Weimerskirch, 2005). Using multiyear satellite tracking data and near‐real‐time environmental data, we developed a species distribution model to identify the oceanographic conditions that characterize and predict foraging habitat for lactating sea lions from the two largest rookeries in the CCS.

The foraging behavior of many central‐place foragers has evolved to repeatedly exploit areas within proximity to breeding grounds where resources may be, to some degree, spatiotemporally predictable, as they offer higher, more efficient levels of energy acquisition and thus maternal provisioning (Baylis, Page, McKenzie, & Goldsworthy, 2012; Bonadonna, Lea, Dehorter, & Guinet, 2001; Chilvers, 2008; Irons, 1998; Lowther, Harcourt, Hamer, & Goldsworthy, 2011). For female California sea lions, we found relationships with static and dynamic environmental covariates that suggest they repeatedly target areas of enhanced productivity. Sea lions preferentially selected cold SST (<14°C), shallow depths, and elevated chlorophyll‐a values, common proxies for upwelling along the continental shelf, where tightly coupled biophysical processes drive the development of a robust food web along central and southern California (Ainley, Sydeman, Parrish, & Lenarz, 1993; Sydeman & Allen, 1999). Coastal upwelling offers seasonally predictable resources on broad spatial scales and has been shown to influence the distribution and abundance of top predators, including several pinniped species off the California coast (Sydeman & Allen, 1999). As California sea lions are known to be shallow water, epipelagic foragers (Feldkamp et al., 1989; Kuhn & Costa, 2014), the combination of shallow depths and upwelling of cold, nutrient waters along the shelf would provide seasonally reliable prey resources. Results from our spatial predictions identified a high degree of suitability close to the breeding colonies and along the California coast, suggesting a strong preference for the nearest predictable and most profitable areas for nursing females.

Interestingly, we found positive associations with metrics of mesoscale activity (e.g., eddy kinetic energy and sea‐level anomalies), suggestive of shelf‐break and offshore foraging, where physical convergence processes can serve as useful foraging patches for top predators due to the aggregation of resources (Bailleul, Cotté, & Guinet, 2010; Hyrenbach, Forney, & Dayton, 2000). Previous top predator studies have documented associations with eddies [e.g., seabirds, Yen et al. (2006); sea turtles, Polovina et al. (2006); and other pinniped species, Fadely, Robson, Sterling, Greig, and Call (2005); Ream, Sterling, and Loughlin (2005)]. While these features are more ephemeral in nature, their persistence in the CCS has been well documented (Batteen, 1997; Lynn & Simpson, 1987; Strub & James, 2000). To our knowledge, this is the first study to detect an association between California sea lion foraging habitat and mesoscale activity. Sea lions are known to display extensive intraspecies variability in their at‐sea movements, behaviors, and distributions (Kuhn & Costa, 2014; McHuron et al., 2016; Melin et al., 2008). This is especially true during lactation, as females must continually expand and adjust their foraging behavior in response to prey movements (Melin et al., 2008) and direct competition for resources under a restricted range and large population size. While finer‐scale studies are necessary to explore an association with eddies in the CCS, it is possible that sea lions may utilize these mesoscale features for foraging opportunities while at sea.

Our modeling approach demonstrates preferred habitat for lactating sea lions is broadly associated with the continental shelf, but exhibits a degree of spatial modulation across years, most notably during basin‐wide environmental perturbations. Considerable studies have documented interannual changes in lactating female foraging behavior in response to El Niño Southern Oscillation (ENSO) events (Costa, Antonelis, & DeLong, 1991; DeLong et al., 1991; Kuhn & Costa, 2014; Melin et al., 2008; Trillmich et al., 1991); however, our ability to broadly identify spatially explicit changes has been limited. For example, higher than normal upwelling and subsequent high productivity during the 2007–2008 La Niña winter provided the most spatially robust habitat availability for sea lions throughout our study period. By the winter of 2008–2009, La Niña conditions had weakened; however, central and northern California maintained stronger than normal upwelling (McClatchie et al., 2009). This most likely drove prey resources north, thereby weakening habitat suitability closest to the breeding colonies. As a result, females would have had to travel farther north to reach access profitable habitat.

Our findings suggest that, during El Niño events, females may face serious limitations in habitat accessibility due to the spatial constraints of the breeding colony. Warmer sea surface temperatures, reduced productivity, and elevated SLA have been associated with reduced food availability in the Southern California Bight (Schwing et al., 2006; Thomas & Brickley, 2006; Trillmich et al., 1991). Suboptimal conditions require females to alter foraging and attendance patterns, with females moving further offshore and north of Monterey Bay to find food (Kuhn & Costa, 2014; Melin et al., 2008), which may result in pup abandonment (Melin et al., 2008). Our model predictions during the 2004–2005 winter reflected this pattern, as a weak El Niño appeared to reduce productivity and prey resources within the nearshore environment (Kuhn & Costa, 2014), shifting habitat suitability away from the southern California coast and north of Monterey Bay. This displacement is consistent with previous studies that identified an increase in adult female abundance in central and northern California during El Niños in response to better foraging conditions (Lowry & Forney, 2005; Sydeman & Allen, 1999).

Although our model predictions extend from 2003 to 2009, the distribution patterns and feature associations we describe here can be used to track shifts in distribution under future ocean conditions. In a warming ocean, alterations to suitable habitat—smaller in extent and further from the breeding colonies—may have severe consequences on the population dynamics of this species. Prolonged environmental change will result in increased energetic costs and decreased reproductive success with potentially long‐term population declines (Hazen et al., 2012; Lowry et al., 2017; McHuron, Mangel, Schwarz, & Costa, 2017; Melin et al., 2008, 2012). Recent studies have documented an increasing number of pups born on Año Nuevo Island, an important central California haul‐out site that supports a small breeding population (Lowry et al., 2017; McHuron, Block, & Costa, 2017). However, there is limited space for the population to expand northwards, as the Channel Islands represent the only islands with enough available space to support such large breeding rookeries under a growing population.

Habitat loss and large sea lion concentrations may lead to increased pressure on coastal fisheries, due to overlap with commercially important prey species, with a potential to impact to top‐down food web dynamics (Lowry & Forney, 2005; Lowry et al., 1991; Weise, Costa, & Kudela, 2006). Furthermore, the relationships we identified have been found in other mobile marine predators that utilize dynamic biophysical features (e.g., upwelling centers, fronts, and eddies) and have important implications for overlap with human use (Maxwell et al., 2013; Scales et al., 2014). Recent research that considers what dynamic habitat use means for ocean resource management has found that while place‐based static management approaches can be used to define general areas of overlap between protected species and human use, our ability to monitor and manage animals in relation to human activities will likely require management approaches that are also dynamic in space and time (Gregr, Lessard, & Harper, 2013; Lewison et al., 2015; Maxwell et al., 2015; Wedding et al., 2016). The predictive models used in this study offer an increased understanding of when and where potential conflicts may arise and reflect the importance of dynamic, spatially explicit conservation and management initiatives for many other marine top predators (Louzao et al., 2011).

Our broadscale modeling approach presented provides information on the population‐level habitat associations for females from the two largest California sea lion breeding colonies in the California Current System. However, some caveats must be considered, as foraging habits and at‐sea distribution can vary by season and life history stage. For lactating females, distribution may be closer to the rookery at different times of year, specifically during the breeding season (June–July). During this time, females give birth and their foraging trips are even more constrained by pup age. Our satellite observations did not include this period; therefore, we caution extrapolation of model predictions to other data‐limited times of the year. Second, separate species distribution models should be constructed for nonlactating females, as are free to disperse away from the rookery to exploit productive areas (Melin et al., 2000). Finally, habitat selection may be colony‐specific. While core residency for both colonies indicated proximity of lactating females to breeding grounds and some overlap in spatial distribution, San Miguel Island females were more likely to move north near Monterey Bay, whereas only San Nicolas Island individuals used the Southern California Bight (Figure 1b,c). This spatial foraging segregation may be a mechanism to reduce intraspecific competition between two breeding colonies (Kuhn & Costa, 2014; McHuron et al., 2016), but also may reflect spatial constraints associated with lactation, as San Nicolas is approximately 120 km southeast of San Miguel (Kuhn & Costa, 2014). Future work may include finer‐scale, colony‐specific, or behavior‐specific models that may capture habitat preference more relevant to specific colonies or individual behaviors. Additional tagging studies may help capture suitable habitat for all colonies within the Southern California Bight, including Santa Barbara Island and San Clemente Island. However, for the purposes of understanding the environmental drivers that influence distribution on a population level, this study represents an important first step in modeling spatially explicit habitat use for this species. Our findings demonstrate the utility of a marine species distribution model as a novel approach for identifying changes in central‐place forager habitat, with important implications at the population level. An increased understanding of habitat use can not only improve our ability to monitor and predict future shifts in distribution as a function environmental variability, but also serve in the context of species protection and fisheries management.

## CONFLICT OF INTEREST

None declared.

## AUTHOR CONTRIBUTION

DB, SF, KS, EH, SB, EM, and EB contributed to research design and data analysis. SM, EM, PR, and CK conducted fieldwork and collected the data. DC, SM, EH, SB, LC, and RL were responsible for funding application. DB wrote the first draft. All authors contributed to draft revisions and approved the final version of the manuscript.

## References

[ece33827-bib-0001] Aarts, G. , MacKenzie, M. , McConnell, B. , Fedak, M. , & Matthiopoulos, J. (2008). Estimating space‐use and habitat preference from wildlife telemetry data. Ecography, 31, 140–160. https://doi.org/10.1111/j.2007.0906-7590.05236.x

[ece33827-bib-0002] Ainley, D. G. , Sydeman, W. , Parrish, R. , & Lenarz, W. (1993). Oceanic factors influencing distribution of young rockfish (Sebastes) in central California: A predator's perspective. California Cooperative Oceanic Fisheries Investigations Reports, 34, 133–139.

[ece33827-bib-0003] Antonelis, G. A. , Stewart, B. S. , & Perryman, W. F. (1990). Foraging characteristics of female northern fur seals (*Callorhinus ursinus*) and California sea lions (*Zalophus californianus*). Canadian Journal of Zoology, 68, 150–158. https://doi.org/10.1139/z90-022

[ece33827-bib-0004] Arthur, B. , Hindell, M. , Bester, M. , De Bruyn, P. N. , Trathan, P. , Goebel, M. , & Lea, M.‐A. (2017). Winter habitat predictions of a key Southern Ocean predator, the Antarctic fur seal (*Arctocephalus gazella*). Deep Sea Research Part II: Topical Studies in Oceanography, 140, 171–181. https://doi.org/10.1016/j.dsr2.2016.10.009

[ece33827-bib-0005] Austin, D. , Bowen, W. , & McMillan, J. (2004). Intraspecific variation in movement patterns: Modeling individual behaviour in a large marine predator. Oikos, 105, 15–30. https://doi.org/10.1111/j.0030-1299.1999.12730.x

[ece33827-bib-0006] Bailey, H. , Fossette, S. , Bograd, S. J. , Shillinger, G. L. , Swithenbank, A. M. , Georges, J. Y. , … Hays, G. C. (2012). Movement patterns for a critically endangered species, the leatherback turtle (*Dermochelys coriacea*), linked to foraging success and population status. PLoS One, 7, e36401 https://doi.org/10.1371/journal.pone.0036401 22615767 10.1371/journal.pone.0036401 PMC3354004

[ece33827-bib-0007] Bailey, H. , Shillinger, G. , Palacios, D. , Bograd, S. , Spotila, J. , Paladino, F. , & Block, B. (2008). Identifying and comparing phases of movement by leatherback turtles using state‐space models. Journal of Experimental Marine Biology and Ecology, 356, 128–135. https://doi.org/10.1016/j.jembe.2007.12.020

[ece33827-bib-0008] Bailleul, F. , Cotté, C. , & Guinet, C. (2010). Mesoscale eddies as foraging area of a deep‐diving predator, the southern elephant seal. Marine Ecology Progress Series, 408, 251–264. https://doi.org/10.3354/meps08560

[ece33827-bib-0009] Barbet‐Massin, M. , Jiguet, F. , Albert, C. H. , & Thuiller, W. (2012). Selecting pseudo‐absences for species distribution models: How, where and how many? Methods in Ecology and Evolution, 3, 327–338. https://doi.org/10.1111/j.2041-210X.2011.00172.x

[ece33827-bib-0010] Batteen, M. L. (1997). Wind‐forced modeling studies of currents, meanders, and eddies in the California Current system. Journal of Geophysical Research: Oceans, 102, 985–1010. https://doi.org/10.1029/96JC02803

[ece33827-bib-0011] Baylis, A. M. , Page, B. , McKenzie, J. , & Goldsworthy, S. D. (2012). Individual foraging site fidelity in lactating New Zealand fur seals: Continental shelf vs. oceanic habitats. Marine Mammal Science, 28, 276–294. https://doi.org/10.1111/j.1748-7692.2011.00487.x

[ece33827-bib-0012] Beeson, M. J. , & Hanan, D. A. (1996). An evaluation of pinniped‐fishery interactions in California. Report to the Pacific States Marine Fisheries Commission, 46.

[ece33827-bib-0013] Bonadonna, F. , Lea, M.‐A. , Dehorter, O. , & Guinet, C. (2001). Foraging ground fidelity and route‐choice tactics of a marine predator: The Antarctic fur seal *Arctocephalus gazella* . Marine Ecology Progress Series, 223, 287–297. https://doi.org/10.3354/meps223287

[ece33827-bib-0014] Bost, C.‐A. , Cotté, C. , Bailleul, F. , Cherel, Y. , Charrassin, J.‐B. , Guinet, C. , … Weimerskirch, H. (2009). The importance of oceanographic fronts to marine birds and mammals of the southern oceans. Journal of Marine Systems, 78, 363–376. https://doi.org/10.1016/j.jmarsys.2008.11.022

[ece33827-bib-0015] Breed, G. A. , Costa, D. P. , Goebel, M. E. , & Robinson, P. W. (2011). Electronic tracking tag programming is critical to data collection for behavioral time‐series analysis. Ecosphere, 2, art10.

[ece33827-bib-0016] Breed, G. A. , Costa, D. P. , Jonsen, I. D. , Robinson, P. W. , & Mills‐Flemming, J. (2012). State‐space methods for more completely capturing behavioral dynamics from animal tracks. Ecological Modelling, 235, 49–58. https://doi.org/10.1016/j.ecolmodel.2012.03.021

[ece33827-bib-0017] Buckley, L. B. , Urban, M. C. , Angilletta, M. J. , Crozier, L. G. , Rissler, L. J. , & Sears, M. W. (2010). Can mechanism inform species' distribution models? Ecology Letters, 13, 1041–1054. 20482574 10.1111/j.1461-0248.2010.01479.x

[ece33827-bib-0018] Calenge, C. (2011). Home range estimation in R: The adehabitatHR package. Saint Benoist, Auffargis, France: Office national de la classe et de la faune sauvage.

[ece33827-bib-0019] Calenge, C. (2015). Analysis of animal movements in R: The adehabitatLT Package. Vienna, Austria: R Foundation for Statistical Computing.

[ece33827-bib-0020] Calenge, C. , Dray, S. , & Royer‐Carenzi, M. (2009). The concept of animals' trajectories from a data analysis perspective. Ecological Informatics, 4, 34–41. https://doi.org/10.1016/j.ecoinf.2008.10.002

[ece33827-bib-0021] Carretta, J. V. , & Chivers, S. J. (2004). Preliminary estimates of marine mammal mortality and biological sampling of cetaceans in California gillnet fisheries for 2003. In Paper SC/56/SM1 presented to the IWC Scientific Committee, June 2004 (unpublished).[Available from Southwest Fisheries Science Center, National Marine Fisheries Service, 8604 La Jolla Shores Drive, La Jolla, CA 92037, USA]

[ece33827-bib-0022] Carretta, J. , Forney, K. , & Oleson, E. (2011) U.S. Pacific marine mammal stock assessments: 2011. *U.S. Department of Commerce, NOAA Technical Memorandum*, NOAA‐TM‐NMFS‐SWFSC‐488, 356.

[ece33827-bib-0023] Carretta, J. V. , Oleson, E. , Baker, J. , Weller, D. W. , Lang, A. R. , Forney, K. A. , … Brownell, R. L. Jr (2016). US Pacific draft marine mammal stock assessments: 2015. NOAA Technical Memorandum NMFS‐SWFSC‐561. La Jolla, CA: National Oceanic and Atmospheric Administration.

[ece33827-bib-0024] Carvalho, S. B. , Brito, J. C. , Crespo, E. G. , Watts, M. E. , & Possingham, H. P. (2011). Conservation planning under climate change: Toward accounting for uncertainty in predicted species distributions to increase confidence in conservation investments in space and time. Biological Conservation, 144, 2020–2030. https://doi.org/10.1016/j.biocon.2011.04.024

[ece33827-bib-0025] Cayula, J.‐F. , & Cornillon, P. (1992). Edge detection algorithm for SST images. Journal of Atmospheric and Oceanic Technology, 9, 67–80. https://doi.org/10.1175/1520-0426(1992)009<0067:EDAFSI>2.0.CO;2

[ece33827-bib-0026] Chavez, F. P. , & Messié, M. (2009). A comparison of eastern boundary upwelling ecosystems. Progress in Oceanography, 83, 80–96. https://doi.org/10.1016/j.pocean.2009.07.032

[ece33827-bib-0027] Checkley, D. M. , & Barth, J. A. (2009). Patterns and processes in the California Current System. Progress in Oceanography, 83, 49–64. https://doi.org/10.1016/j.pocean.2009.07.028

[ece33827-bib-0028] Chilvers, B. (2008). Foraging site fidelity of lactating New Zealand sea lions. Journal of Zoology, 276, 28–36. https://doi.org/10.1111/j.1469-7998.2008.00463.x

[ece33827-bib-0029] Costa, D. P. (1991). Reproductive and foraging energetics of pinnipeds: Implications for life history patterns In Renouf D. (Ed.), The behaviour of pinnipeds (pp. 300–344). Chapman and Hall, London, UK: Springer https://doi.org/10.1007/978-94-011-3100-1

[ece33827-bib-0030] Costa, D. P. (2007). A conceptual model of the variation in parental attendance in response to environmental fluctuation: Foraging energetics of lactating sea lions and fur seals. Aquatic Conservation: Marine and Freshwater Ecosystems, 17, S44–S52. https://doi.org/10.1002/(ISSN)1099-0755

[ece33827-bib-0031] Costa, D. , Antonelis, G. , & DeLong, R. (1991). Effects of El Niño on the foraging energetics of the California sea lion In Trillmich F. and Ono K. (Eds.), Pinnipeds and El Niño Pinnipeds and El Nino: Responses to Environmental Stress (pp. 156–165). Springer‐Verlag, Berlin, Germany: Springer https://doi.org/10.1007/978-3-642-76398-4

[ece33827-bib-0032] Croll, D. A. , Marinovic, B. , Benson, S. , Chavez, F. P. , Black, N. , Ternullo, R. , & Tershy, B. R. (2005). From wind to whales: Trophic links in a coastal upwelling system. Marine Ecology Progress Series, 289, 117–130. https://doi.org/10.3354/meps289117

[ece33827-bib-0033] Dambach, J. , & Rödder, D. (2011). Applications and future challenges in marine species distribution modeling. Aquatic Conservation: Marine and Freshwater Ecosystems, 21, 92–100. https://doi.org/10.1002/aqc.1160

[ece33827-bib-0034] DeLong, R. , Antonelis, G. , Oliver, C. , Stewart, B. , Lowry, M. , & Yochem, P. (1991). Effects of the 1982–83 El Nino on several population parameters and diet of California sea lions on the California Channel Islands In Trillmich F. and Ono K. (Eds.), Pinnipeds and El Nino: Responses to Environmental Stress (pp. 166–172). Springer‐Verlag, Berlin, Germany: Springer https://doi.org/10.1007/978-3-642-76398-4

[ece33827-bib-0035] Eguchi, T. , Benson, S. R. , Foley, D. G. , & Forney, K. A. (2017). Predicting overlap between drift gillnet fishing and leatherback turtle habitat in the California Current Ecosystem. Fisheries Oceanography, 26, 17–33. https://doi.org/10.1111/fog.12181

[ece33827-bib-0036] Elith, J. , & Leathwick, J. R. (2009). Species distribution models: Ecological explanation and prediction across space and time. Annual Review of Ecology, Evolution, and Systematics, 40, 677–697. https://doi.org/10.1146/annurev.ecolsys.110308.120159

[ece33827-bib-0037] Fadely, B. S. , Robson, B. W. , Sterling, J. T. , Greig, A. , & Call, K. A. (2005). Immature Steller sea lion (*Eumetopias jubatus*) dive activity in relation to habitat features of the eastern Aleutian Islands. Fisheries Oceanography, 14, 243–258. https://doi.org/10.1111/j.1365-2419.2005.00379.x

[ece33827-bib-0038] Feldkamp, S. D. , DeLong, R. L. , & Antonelis, G. A. (1989). Diving patterns of California sea lions, *Zalophus californianus* . Canadian Journal of Zoology, 67, 872–883. https://doi.org/10.1139/z89-129

[ece33827-bib-0039] Ficetola, G. F. , Pennati, R. , & Manenti, R. (2013). Spatial segregation among age classes in cave salamanders: Habitat selection or social interactions? Population Ecology, 55, 217–226. https://doi.org/10.1007/s10144-012-0350-5

[ece33827-bib-0040] Goldstein, T. , Johnson, S. , Phillips, A. , Hanni, K. , & Fauquier, D. (1999). Human‐related injuries observed in live stranded pinnipeds along the. Aquatic Mammals, 25, 43–51.

[ece33827-bib-0041] Gregr, E. J. , Lessard, J. , & Harper, J. (2013). A spatial framework for representing nearshore ecosystems. Progress in Oceanography, 115, 189–201. https://doi.org/10.1016/j.pocean.2013.05.028

[ece33827-bib-0042] Guisan, A. , & Zimmermann, N. E. (2000). Predictive habitat distribution models in ecology. Ecological Modelling, 135, 147–186. https://doi.org/10.1016/S0304-3800(00)00354-9

[ece33827-bib-0043] Hastie, T. J. , & Tibshirani, R. J. (1990). Generalized additive models. John Wiley & Sons, Inc.: CRC Press.

[ece33827-bib-0044] Hazen, E. L. , Jorgensen, S. , Rykaczewski, R. R. , Bograd, S. J. , Foley, D. G. , Jonsen, I. D. , … Block, B. A. (2012). Predicted habitat shifts of Pacific top predators in a changing climate. Nature Climate Change, 3, 234–238. https://doi.org/10.1038/nclimate1686

[ece33827-bib-0045] Hazen, E. L. , Palacios, D. , Forney, K. , Howell, E. A. , Becker, E. A. , Hoover, A. L. , … Bailey, H. (2016). WhaleWatch: A dynamic management tool for predicting blue whale density in the California Current. Journal of Applied Ecology, 54, 1415–1428.

[ece33827-bib-0046] Hobday, A. J. , Hartog, J. R. , Spillman, C. M. , Alves, O. , & Hilborn, R. (2011). Seasonal forecasting of tuna habitat for dynamic spatial management. Canadian Journal of Fisheries and Aquatic Sciences, 68, 898–911. https://doi.org/10.1139/f2011-031

[ece33827-bib-0047] Hooker, S. K. , Cañadas, A. , Hyrenbach, K. D. , Corrigan, C. , Polovina, J. J. , & Reeves, R. R. (2011). Making protected area networks effective for marine top predators. Endangered Species Research, 13, 203–218. https://doi.org/10.3354/esr00322

[ece33827-bib-0048] Hyrenbach, K. D. , Forney, K. A. , & Dayton, P. K. (2000). Marine protected areas and ocean basin management. Aquatic Conservation: Marine and Freshwater Ecosystems, 10, 437–458. https://doi.org/10.1002/(ISSN)1099-0755

[ece33827-bib-0049] Irons, D. B. (1998). Foraging area fidelity of individual seabirds in relation to tidal cycles and flock feeding. Ecology, 79, 647–655. https://doi.org/10.1890/0012-9658(1998)079[0647:FAFOIS]2.0.CO;2

[ece33827-bib-0050] Jonsen, I. D. , Flemming, J. M. , & Myers, R. A. (2005). Robust state–space modeling of animal movement data. Ecology, 86, 2874–2880. https://doi.org/10.1890/04-1852

[ece33827-bib-0051] Kappes, M. A. , Shaffer, S. A. , Tremblay, Y. , Foley, D. G. , Palacios, D. M. , Robinson, P. W. , … Costa, D. P. (2010). Hawaiian albatrosses track interannual variability of marine habitats in the North Pacific. Progress in Oceanography, 86, 246–260. https://doi.org/10.1016/j.pocean.2010.04.012

[ece33827-bib-0052] Kuhn, C. E. , & Costa, D. P. (2014). Interannual variation in the at‐sea behavior of California sea lions (Zalophus californianus). Marine Mammal Science, 30, 1297–1319. https://doi.org/10.1111/mms.12110

[ece33827-bib-0053] Lewison, R. , Hobday, A. J. , Maxwell, S. , Hazen, E. , Hartog, J. R. , Dunn, D. C. , … Barnes, M. (2015). Dynamic ocean management: Identifying the critical ingredients of dynamic approaches to ocean resource management. BioScience, 65, 486–498.

[ece33827-bib-0054] Louzao, M. , Pinaud, D. , Peron, C. , Delord, K. , Wiegand, T. , & Weimerskirch, H. (2011). Conserving pelagic habitats: Seascape modelling of an oceanic top predator. Journal of Applied Ecology, 48, 121–132. https://doi.org/10.1111/j.1365-2664.2010.01910.x

[ece33827-bib-0055] Lowry, M. S. , & Carretta, J. V. (1999) Market squid (Loligo opalescens) in the diet of California sea lions (Zalophus californianus) in southern California (1981–1995). *California Cooperative Oceanic Fisheries Investigations Report*, 196–207.

[ece33827-bib-0056] Lowry, M. S. , & Forney, K. A. (2005). Abundance and distribution of California sea lions (*Zalophus californianus*) in central and northern California during 1998 and summer 1999. Fishery Bulletin, 103, 331–343.

[ece33827-bib-0057] Lowry, M. S. , Melin, S. R. , & Laake, J. L. (2017). Breeding season distribution and population growth of California sea lions, Zalophus californianus, in the United States during 1964–2014. NOAA Technical Memorandum NMFS‐SWFSC‐574. La Jolla, CA: National Oceanic and Atmospheric Administration.

[ece33827-bib-0058] Lowry, M. S. , Stewart, B. S. , Heath, C. B. , Yochem, P. K. , & Francis, M. (1991). Seasonal and annual variability in the diet of California sea lions, *Zalophus californianus*, at San Nicolas Island, California, 1981–86. Fishery Bulletin, 89, 331–336.

[ece33827-bib-0059] Lowther, A. , Harcourt, R. G. , Hamer, D. , & Goldsworthy, S. (2011). Creatures of habit: Foraging habitat fidelity of adult female Australian sea lions. Marine Ecology Progress Series, 443, 249–263. https://doi.org/10.3354/meps09392

[ece33827-bib-0060] Lynn, R. J. , & Simpson, J. J. (1987). The California current system: The seasonal variability of its physical characteristics. Journal of Geophysical Research: Oceans, 92, 12947–12966. https://doi.org/10.1029/JC092iC12p12947

[ece33827-bib-0061] Matthiopoulos, J. , Harwood, J. , & Thomas, L. (2005). Metapopulation consequences of site fidelity for colonially breeding mammals and birds. Journal of Animal Ecology, 74, 716–727. https://doi.org/10.1111/j.1365-2656.2005.00970.x

[ece33827-bib-0062] Maxwell, S. M. , Hazen, E. L. , Bograd, S. J. , Halpern, B. S. , Breed, G. A. , Nickel, B. , … Dutton, P. H. (2013). Cumulative human impacts on marine predators. Nature Communications, 4, 2688. 10.1038/ncomms3688 24162104

[ece33827-bib-0063] Maxwell, S. M. , Hazen, E. L. , Lewison, R. L. , Dunn, D. C. , Bailey, H. , Bograd, S. J. , … Bennett, M. (2015). Dynamic ocean management: Defining and conceptualizing real‐time management of the ocean. Marine Policy, 58, 42–50. https://doi.org/10.1016/j.marpol.2015.03.014

[ece33827-bib-0064] McClatchie, S. , Charter, R. , Watson, W. , Lo, N. , Hill, K. , Manzano‐Sarabia, M. , … Schwing, F. B. (2009). State of the California Current, Spring 2008–2009: Cold conditions drive regional differences in coastal production. Progress Report No. 50 (pp.43‐68). California Cooperative Oceanic Fishes Investigations

[ece33827-bib-0065] McClatchie, S. , Field, J. , Thompson, A. R. , Gerrodette, T. , Lowry, M. , Fiedler, P. C. , … Vetter, R. D. (2016). Food limitation of sea lion pups and the decline of forage off central and southern California. Open Science, 3, 150628. 10.1098/rsos.150628 PMC4821262 27069651

[ece33827-bib-0066] McDonald, B. I. , & Ponganis, P. J. (2013). Insights from venous oxygen profiles: Oxygen utilization and management in diving California sea lions. Journal of Experimental Biology, 216, 3332–3341. https://doi.org/10.1242/jeb.085985 23926312 10.1242/jeb.085985

[ece33827-bib-0067] McHuron, E. A. , Block, B. A. , & Costa, D. P. (2017). Movements and dive behaviour of juvenile California sea lions from Año Nuevo Island. Marine Mammal Science, 34.1, 238–249.

[ece33827-bib-0068] McHuron, E. , Mangel, M. , Schwarz, L. , & Costa, D. (2017). Energy and prey requirements of California sea lions under variable environmental conditions. Marine Ecology Progress Series, 567, 235–247. https://doi.org/10.3354/meps12041

[ece33827-bib-0069] McHuron, E. , Robinson, P. , Simmons, S. , Kuhn, C. , Fowler, M. , & Costa, D. (2016). Foraging strategies of a generalist marine predator inhabiting a dynamic environment. Oecologia, 182, 995–1005. https://doi.org/10.1007/s00442-016-3732-0 27651228 10.1007/s00442-016-3732-0

[ece33827-bib-0070] Melin, S. , DeLong, R. , & Siniff, D. (2008). The effects of El Niño on the foraging behavior of lactating California sea lions (*Zalophus californianus californianus*) during the nonbreeding season. Canadian Journal of Zoology, 86, 192–206. https://doi.org/10.1139/Z07-132

[ece33827-bib-0071] Melin, S. R. , Delong, R. L. , Thomason, J. R. , & Vanblaricom, G. R. (2000). Attendance patterns of California sea lion (*Zalophus californianus*) females and pups during the non‐breeding season at San Miguel Island. Marine Mammal Science, 16, 169–185. https://doi.org/10.1111/j.1748-7692.2000.tb00911.x

[ece33827-bib-0072] Melin, S. R. , Orr, A. J. , Harris, J. D. , Laake, J. L. , & DeLong, R. L. (2012). California sea lions: An indicator for integrated ecosystem assessment of the California current system. California Cooperative Oceanic Fisheries Investigations Reports, 53, 140–152.

[ece33827-bib-0073] NMFS (1997). National Marine Fisheries Service. Impacts of California sea lions and Pacific harbor seals on salmonids and the coastal ecosystems of Washington, Oregon, and California. NOAA Technial Memorandum NMFS‐NWFSC‐28.

[ece33827-bib-0074] Ono, K. A. , Boness, D. J. , & Oftedal, O. T. (1987). The effect of a natural environmental disturbance on maternal investment and pup behavior in the California sea lion. Behavioral Ecology and Sociobiology, 21, 109–118. https://doi.org/10.1007/BF02395438

[ece33827-bib-0075] Orians, G. H. , & Pearson, N. E. (1979). On the theory of central place foraging In Horn D.J., Mitchell R.D. and Straits G.R. (Eds.), Analysis of ecological systems (pp. 155–177). Columbus, OH: Ohio State University Press.

[ece33827-bib-0076] Orr, A. , VanBlaricom, G. , DeLong, R. , Cruz‐Escalona, V. H. , & Newsome, S. (2011). Intraspecific comparison of diet of California sea lions (*Zalophus californianus*) assessed using fecal and stable isotope analyses. Canadian Journal of Zoology, 89, 109–122. https://doi.org/10.1139/Z10-101

[ece33827-bib-0077] Pinaud, D. , & Weimerskirch, H. (2005). Scale‐dependent habitat use in a long‐ranging central place predator. Journal of Animal Ecology, 74, 852–863. https://doi.org/10.1111/j.1365-2656.2005.00984.x

[ece33827-bib-0078] Polovina, J. , Uchida, I. , Balazs, G. , Howell, E. A. , Parker, D. , & Dutton, P. (2006). The Kuroshio extension bifurcation region: A pelagic hotspot for juvenile loggerhead sea turtles. Deep Sea Research Part II: Topical Studies in Oceanography, 53, 326–339. https://doi.org/10.1016/j.dsr2.2006.01.006

[ece33827-bib-0079] R Core Team (2016). R: A language and environment for statistical computing. Vienna, Austria: R Foundation for Statistical Computing http://www.R-project.org/

[ece33827-bib-0080] Ream, R. R. , Sterling, J. T. , & Loughlin, T. R. (2005). Oceanographic features related to northern fur seal migratory movements. Deep Sea Research Part II: Topical Studies in Oceanography, 52, 823–843. https://doi.org/10.1016/j.dsr2.2004.12.021

[ece33827-bib-0081] Redfern, J. , Ferguson, M. , Becker, E. , Hyrenbach, K. , Good, C. , Barlow, J. , … Ballance, L. (2006). Techniques for cetacean–habitat modeling. Marine Ecology Progress Series, 310, 271–295. https://doi.org/10.3354/meps310271

[ece33827-bib-0082] Roman, J. , Altman, I. , Dunphy‐Daly, M. M. , Campbell, C. , Jasny, M. , & Read, A. J. (2013). The marine mammal protection act at 40: Status, recovery, and future of US marine mammals. Annals of the New York Academy of Sciences, 1286, 29–49. https://doi.org/10.1111/nyas.12040 23521536 10.1111/nyas.12040

[ece33827-bib-0083] Rosenberg, D. K. , & McKelvey, K. S. (1999). Estimation of habitat selection for central‐place foraging animals. The Journal of Wildlife Management, 63, 1028–1038. https://doi.org/10.2307/3802818

[ece33827-bib-0084] Scales, K. L. , Miller, P. I. , Hawkes, L. A. , Ingram, S. N. , Sims, D. W. , & Votier, S. C. (2014). On the Front Line: Frontal zones as priority at‐sea conservation areas for mobile marine vertebrates. Journal of Applied Ecology, 51, 1575–1583. https://doi.org/10.1111/1365-2664.12330

[ece33827-bib-0085] Scales, K. L. , Schorr, G. S. , Hazen, E. L. , Bograd, S. J. , Miller, P. I. , Andrews, R. D. , … Falcone, E. A. (2017). Should I stay or should I go? Modelling year‐round habitat suitability and drivers of residency for fin whales in the California Current. Diversity and Distributions, 23, 1204–1215. https://doi.org/10.1111/ddi.12611

[ece33827-bib-0086] Schwing, F. B. , Bond, N. A. , Bograd, S. J. , Mitchell, T. , Alexander, M. A. , & Mantua, N. (2006). Delayed coastal upwelling along the US West Coast in 2005: A historical perspective. Geophysical Research Letters, 33, L22S01 https://doi.10.1029/2006GL026911

[ece33827-bib-0087] Schwing, F. , Husby, D. , Garfield, N. , & Tracy, D. (1991). Mesoscale oceanic response to wind events off central California in spring 1989: CTD surveys and AVHRR imagery. California Cooperative Oceanic Fisheries Investigations Report, 32, 47–62.

[ece33827-bib-0088] Simmons, R. A. (2016). ERDDAP. Monterey, CA: NOAA/NMFS/SWFSC/ERD https://coastwatch.pfeg.noaa.gov/erddap

[ece33827-bib-0089] Sing, T. , Sander, O. , Beerenwinkel, N. , & Lengauer, T. (2015). ROCR. *R Package–Visualizing the Performance of Scoring Classifiers* Available online: https://rdrr.io/cran/ROCR/

[ece33827-bib-0090] Skov, H. , Heinänen, S. , Thaxter, C. B. , Williams, A. E. , Lohier, S. , & Banks, A. N. (2016). Real‐time species distribution models for conservation and management of natural resources in marine environments. Marine Ecology Progress Series, 542, 221–234. https://doi.org/10.3354/meps11572

[ece33827-bib-0091] Stewart, B. S. , & Yochem, P. K. (1987). Entanglement of pinnipeds in synthetic debris and fishing net and line fragments at San Nicholas and San Miguel Islands, California, 1978–1986. Marine Pollution Bulletin, 18, 336–339. https://doi.org/10.1016/S0025-326X(87)80021-8

[ece33827-bib-0092] Strub, P. T. , & James, C. (2000). Altimeter‐derived variability of surface velocities in the California Current System: 2. Seasonal circulation and eddy statistics. Deep Sea Research Part II: Topical Studies in Oceanography, 47, 831–870. https://doi.org/10.1016/S0967-0645(99)00129-0

[ece33827-bib-0093] Studwell, A. J. , Hines, E. , Elliott, M. L. , Howar, J. , Holzman, B. , Nur, N. , & Jahncke, J. (2017). Modeling nonresident seabird foraging distributions to inform ocean zoning in Central California. PLoS One, 12, e0169517 https://doi.org/10.1371/journal.pone.0169517 28122001 10.1371/journal.pone.0169517 PMC5266262

[ece33827-bib-0094] Su, N.‐J. , Sun, C.‐L. , Punt, A. E. , Yeh, S.‐Z. , & DiNardo, G. (2011). Modelling the impacts of environmental variation on the distribution of blue marlin, *Makaira nigricans*, in the Pacific Ocean. ICES Journal of Marine Science: Journal du Conseil, 68, 1072–1080. https://doi.org/10.1093/icesjms/fsr028

[ece33827-bib-0095] Sydeman, W. J. , & Allen, S. G. (1999). Pinniped population dynamics in central California: Correlations with sea surface temperature and upwelling indices. Marine Mammal Science, 15, 446–461. https://doi.org/10.1111/j.1748-7692.1999.tb00812.x

[ece33827-bib-0096] Thomas, A. C. , & Brickley, P. (2006). Satellite measurements of chlorophyll distribution during spring 2005 in the California Current. Geophysical Research Letters, 33, L22S05 https://doi.org/10.1029/2006GL026588

[ece33827-bib-0097] Trillmich, F. , Ono, K. , Costa, D. , DeLong, R. , Feldkamp, S. , Francis, J. , … Majluf, P. (1991) The effects of El Nino on pinniped populations in the eastern Pacific In Trillmich F. and Ono K. (Eds.), Pinnipeds and El Niño Pinnipeds and El Nino: Responses to Environmental Stress (pp. 247–270). Springer‐Verlag, Berlin, Germany: Springer https://doi.org/10.1007/978-3-642-76398-4

[ece33827-bib-0098] Villegas‐Amtmann, S. , Atkinson, S. , Paras‐Garcia, A. , & Costa, D. P. (2012). Seasonal variation in blood and muscle oxygen stores attributed to diving behavior, environmental temperature and pregnancy in a marine predator, the California sea lion. Comparative Biochemistry and Physiology Part A: Molecular & Integrative Physiology, 162, 413–420. https://doi.org/10.1016/j.cbpa.2012.04.019 10.1016/j.cbpa.2012.04.019 22561663

[ece33827-bib-0099] Villegas‐Amtmann, S. , Costa, D. P. , Tremblay, Y. , Salazar, S. , & Aurioles‐Gamboa, D. (2008). Multiple foraging strategies in a marine apex predator, the Galapagos sea lion *Zalophus wollebaeki* . Marine Ecology Progress Series, 363, 299–309. https://doi.org/10.3354/meps07457

[ece33827-bib-0100] Villegas‐Amtmann, S. , Simmons, S. E. , Kuhn, C. E. , Huckstadt, L. A. , & Costa, D. P. (2011). Latitudinal range influences the seasonal variation in the foraging behavior of marine top predators. PLoS One, 6, e23166 https://doi.org/10.1371/journal.pone.0023166 21853081 10.1371/journal.pone.0023166 PMC3154271

[ece33827-bib-0101] Wedding, L. , Maxwell, S. , Hyrenbach, D. , Dunn, D. , Roberts, J. , Briscoe, D. , … Halpin, P. (2016). Geospatial approaches to support pelagic conservation planning and adaptive management. Endangered Species Research, 30, 1–9. https://doi.org/10.3354/esr00716

[ece33827-bib-0102] Weise, M. J. , Costa, D. P. , & Kudela, R. M. (2006). Movement and diving behavior of male California sea lion (*Zalophus californianus*) during anomalous oceanographic conditions of 2005 compared to those of 2004. Geophysical Research Letters, 33, L22S10 https://doi.org/10.1029/2006GL027113

[ece33827-bib-0103] Weise, M. J. , & Harvey, J. T. (2005). Impact of the California sea lion (*Zalophus californianus*) on salmon fisheries in Monterey Bay, California. Fishery Bulletin, 103, 685–696.

[ece33827-bib-0104] Weise, M. J. , & Harvey, J. T. (2008). Temporal variability in ocean climate and California sea lion diet and biomass consumption: Implications for fisheries management. Marine Ecology Progress Series, 373, 157–172. https://doi.org/10.3354/meps07737

[ece33827-bib-0105] Willis‐Norton, E. , Hazen, E. L. , Fossette, S. , Shillinger, G. , Rykaczewski, R. R. , Foley, D. G. , … Bograd, S. J. (2015). Climate change impacts on leatherback turtle pelagic habitat in the Southeast Pacific. Deep Sea Research Part II: Topical Studies in Oceanography, 113, 260–267. https://doi.org/10.1016/j.dsr2.2013.12.019

[ece33827-bib-0106] Wood, S. (2006) Generalized additive models: An introduction with R. Chapman Hall. CRC Press. Boca Raton, FL: CRC press.

[ece33827-bib-0107] Wood, S. , & Scheipl, F. (2013). gamm4: Generalized additive mixed models using mgcv and lme4. In. R package version 0.2‐2, http://CRAN.R-project.org/package=gamm4

[ece33827-bib-0108] Yen, P. , Sydeman, W. , Bograd, S. , & Hyrenbach, K. (2006). Spring‐time distributions of migratory marine birds in the southern California Current: Oceanic eddy associations and coastal habitat hotspots over 17 years. Deep Sea Research Part II: Topical Studies in Oceanography, 53, 399–418. https://doi.org/10.1016/j.dsr2.2006.01.013

[ece33827-bib-0109] Zuur, A. F. , Ieno, E. N. , Walker, N. J. , Saveliev, A. A. , & Smith, G. M. (2009). Zero‐truncated and zero‐inflated models for count data In Gail M., Krickeberg K., Samet J.M., Tsiatis A., and Wong W. (Eds.), Mixed effects models and extensions in ecology with R (pp. 261–293). Statistics for Biology and Health. Springer, New York, NY: Springer https://doi.org/10.1007/978-0-387-87458-6

[ece33827-bib-0110] Zydelis, R. , Lewison, R. L. , Shaffer, S. A. , Moore, J. E. , Boustany, A. M. , Roberts, J. J. , … Crowder, L. B. (2011). Dynamic habitat models: Using telemetry data to project fisheries bycatch. Proceedings of the Royal Society of London B: Biological Sciences, 278, 3191–3200. https://doi.org/10.1098/rspb.2011.0330 10.1098/rspb.2011.0330 PMC3169031 21429921

