# Supplementary material for: Characterizing habitat suitability for a central‐place forager in a dynamic marine environment
Source: Ecol Evol. 2018 Feb 9;8(5):2788–801. doi: 10.1002/ece3.3827 (PMC5838083; doi:10.1002/ece3.3827)

**SUPPLEMENTAL TABLES**

**Table S1**. Satellite product and spatio-temporal resolution of environmental variables used in the modeling of California sea lion habitat selection.

| **Product** | **Source** | **Spatial Resolution** | **Temporal Resolution** |
| --- | --- | --- | --- |
| Sea-Surface Temperature (SST) | GHRSST/Reynolds OI | 0.25° | 1-day |
| Chlorophyll-a (Chl-a) | SeaWiFS/MODIS | 0.0125° | 8-day |
| Mean sea level anomaly (SLA) | AVISO | 0.25° | 1-day |
| Eddy kinetic energy (EKE) | AVISO | 0.25° | 1-day |
| Seawinds | QuikSCAT/METOP ASCAT | 0.25° | 3-day |
| Bathymetry | ETOPO 1 | 1° | **-** |
| Distance to colony | ETOPO 1 | 1 m | **-** |

**SUPPLEMENTAL FIGURES**

**Figure S1.** Sample comparison of actual sea lion trip (red) and correlated random walk pseudo-absences (blue), Tag ID: 2104007.


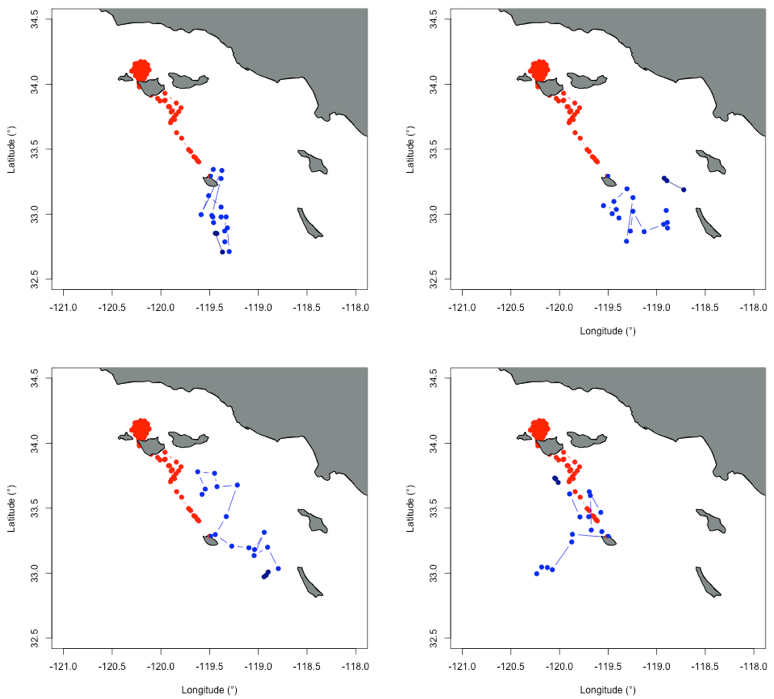


**Figure S2.** Receiver operating curves (ROCs) for final GAMM model run 40 times, with false positive on the x-axis plotted against the true positive on the y-axis. Average AUC = 0.91. A perfect ROC would have a false positive rate of 0 and a true positive rate of 1.


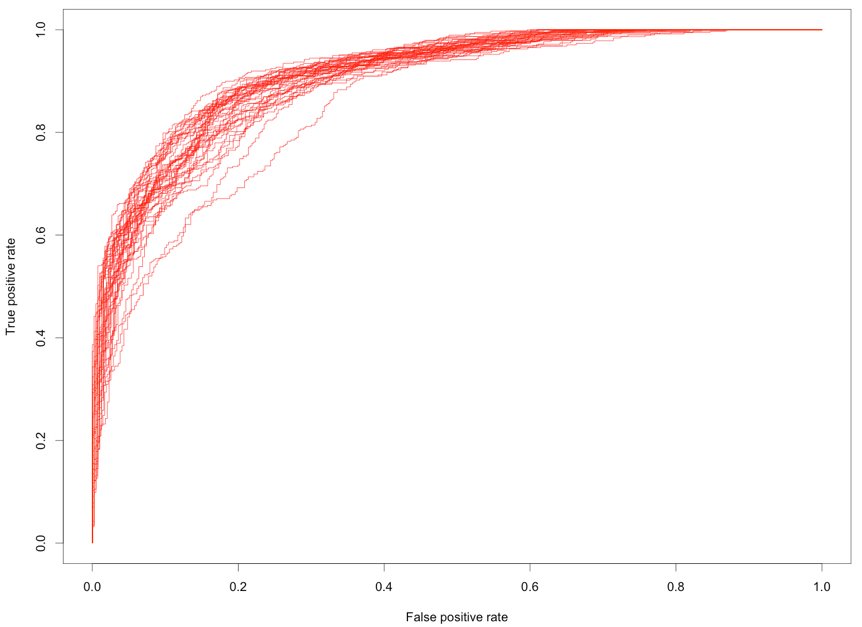

Supplement: Supplementary file 1 [file ECE3-8-2788-s001.docx]
